# The ancestral chromatin landscape of land plants

**DOI:** 10.1111/nph.19311

**Published:** 2023-10-12

**Authors:** Tetsuya Hisanaga, Shuangyang Wu, Peter Schafran, Elin Axelsson, Svetlana Akimcheva, Liam Dolan, Fay‐Wei Li, Frédéric Berger

**Affiliations:** ^1^ Gregor Mendel Institute Austrian Academy of Sciences, Vienna BioCenter Dr. Bohr‐Gasse 3 Vienna 1030 Austria; ^2^ Boyce Thompson Institute Ithaca NY 14853 USA; ^3^ Plant Biology Section Cornell University Ithaca NY 14853 USA

**Keywords:** *Anthoceros agrestis*, bryophytes, chromatin, epigenetic, evolution, *Marchantia polymorpha*

## Abstract

- Recent studies have shown that correlations between chromatin modifications and transcription vary among eukaryotes. This is the case for marked differences between the chromatin of the moss *Physcomitrium patens* and the liverwort *Marchantia polymorpha*. Mosses and liverworts diverged from hornworts, altogether forming the lineage of bryophytes that shared a common ancestor with land plants. We aimed to describe chromatin in hornworts to establish synapomorphies across bryophytes and approach a definition of the ancestral chromatin organization of land plants.
- We used genomic methods to define the 3D organization of chromatin and map the chromatin landscape of the model hornwort *Anthoceros agrestis*.
- We report that nearly half of the hornwort transposons were associated with facultative heterochromatin and euchromatin and formed the center of topologically associated domains delimited by protein coding genes. Transposons were scattered across autosomes, which contrasted with the dense compartments of constitutive heterochromatin surrounding the centromeres in flowering plants.
- Most of the features observed in hornworts are also present in liverworts or in mosses but are distinct from flowering plants. Hence, the ancestral genome of bryophytes was likely a patchwork of units of euchromatin interspersed within facultative and constitutive heterochromatin. We propose this genome organization was ancestral to land plants.

## Introduction

A hallmark of eukaryotes is the association of their genome with nucleosomes composed of 147 bp of DNA wrapped by two heterodimers of histones H2A and H2B and a tetramer of histone H3 and H4 (Malik & Henikoff, 2003; Zhou *et al*., 2019; Talbert & Henikoff, 2021a; Sato *et al*., 2022). A plethora of posttranslational modifications (PTMs) on N‐ and C‐ terminal tails of core histones has been identified including methylation, phosphorylation, and acetylation of H2A, H2B, and H3 that are conserved and found in similar genomic contexts across eukaryotes (Sequeira‐Mendes *et al*., 2014; Allis & Jenuwein, 2016; Grau‐Bové *et al*., 2022; Jamge *et al*., 2022).

Posttranslational modifications signal the position of the transcriptional start sites, gene bodies, and terminators (Gardner *et al*., 2011; Kornberg & Lorch, 2020; Leng *et al*., 2020; Talbert & Henikoff, 2021b; Jamge & Berger, 2022) and are involved in the regulation of cell cycle checkpoints, heterochromatic formation, centromere assembly, DNA replication, DNA repair, and gene transcription among others (Bannister & Kouzarides, 2011; Schwämmle *et al*., 2016).

In euchromatin, trimethylation of lysine 4 on histone H3 (H3K4me3) is a PTM associated with the transcription start sites (TSS) of transcribed genes (Shilatifard, 2012; Leng *et al*., 2020) while H3K36me3 is associated with transcriptional elongation (Bannister *et al*., 2005). Facultative heterochromatin forms nuclear domains marked by H3K27me3, which is deposited by polycomb repressive complex 2 (PRC2) (Schuettengruber & Cavalli, 2009; Gan *et al*., 2015). H3K27me3 is crucial for silencing developmental genes (Müller & Verrijzer, 2009; Del Prete *et al*., 2015; Xiao & Wagner, 2015). By contrast, constitutive heterochromatin is occupied by transposons and is marked by H3K9 methylation in yeast, animals, and flowering plants (Allshire & Madhani, 2018). In yeast and animals, H3K9me3 is bound by heterochromatin protein 1 (HP1), which promotes compaction of heterochromatin and forms a compartment that recruits other heterochromatin interacting proteins (Larson & Narlikar, 2018). In flowering plants, H3K9me2 does not bind to HP1 but instead recruits the plant‐specific DNA methyltransferases CHROMOMETHYLASE (CMT) 2 and 3 (Jamge & Berger, 2022). CMT2/3 deposits methyl groups specifically on cytosines in the CHG context (Du *et al*., 2012). H3K9me2 also recruits the *de novo* DNA methyltransferases DOMAINS REARRANGED METHYLASE 1 (DRM1) and DRM2 (X. Li *et al*., 2018) and DNA methylation is bound by a domain present in the SU(VAR)3–9 HOMOLOG (SUVH) 4/5/6 that methylates H3K9 (X. Li *et al*., 2018). Hence, feedback loops involving H3K9 methylation and DNA methylation maintain heterochromatin in flowering plants. In addition, H3K27me1 is deposited on the histone variant H3.1 at constitutive heterochromatin of *Arabidopsis thaliana* by the specific histone methyltransferases ARABIDOPSIS TRITHORAX‐RELATED 5 (ATXR5) and ATXR6 (Jacob *et al*., 2009).

Although histones PTMs and their function are conserved among angiosperms, recent investigations have begun to show a fairly distinct chromatin landscape in bryophytes, which diverged from vascular plants *c*. 500–480 Ma. Bryophytes comprise three monophyletic groups – hornworts, liverworts, and mosses (Harris *et al*., 2022). Five histone PTMs and DNA methylation have been profiled in the genomes of two model bryophytes: the moss *Physcomitrium patens* (Widiez *et al*., 2014; Yaari *et al*., 2019) and the liverwort *Marchantia polymorpha* (Ikeda *et al*., 2018; Montgomery *et al*., 2020). DNA methylation in the CG context is maintained by an ortholog of the flowering plant METHYLTRANSFERASE 1 (MET1), but other pathways that control DNA methylation in angiosperms are not conserved in bryophytes. In *P*. *patens*, *de novo* DNA methylation depends on DNMT3, which is distinct from DRMs involved in the RNA‐directed DNA methylation (RdDM) pathway in *A*. *thaliana* (Yaari *et al*., 2019). *Marchantia polymorpha* has two CMT‐like proteins, which reside outside the clade containing CMT3 (Bewick *et al*., 2017). In addition, horizontal gene transfer of a prokaryotic N4 methyltransferase led to N4 methylation in sperm of *M*. *polymorpha* (Walker *et al*., 2021). While the enzymes that deposit H3K9 methylation have not been characterized in bryophytes, orthologs of SUVH 4/5/6, which are responsible for the deposition of this PTM in *A*. *thaliana*, are present in bryophytes (Bowman *et al*., 2017). H3K27me1 is also present at heterochromatin in *M*. *polymorpha* (Montgomery *et al*., 2020). While H3K36me3 marks the body of actively transcribed genes in *M*. *polymorpha* and *P*. *patens*, as it does in *A*. *thaliana*, H3K4me3 appears to be associated with repressed genes in *M*. *polymorpha* but not in *P*. *patens* (Widiez *et al*., 2014; Montgomery *et al*., 2020). Forty percent of transposons are covered by H3K27me3 in *M*. *polymorpha* (Montgomery *et al*., 2020), but transposons in *P*. *patens* are mostly covered by H3K9me2 (Widiez *et al*., 2014). Thus, *M*. *polymorpha* and *P*. *patens* may differ in the mechanisms associated with DNA methylation and PTMs and their relationship with transcriptional states, genes, and transposons, and it is not possible to draw an overview of the chromatin landscape common to bryophytes based on these representatives of liverworts and mosses, respectively.

Hornworts form a monophyletic group that diverged before the divergence of liverworts and mosses and thus the traits common to hornworts and mosses or liverworts are hypothetically representative of the ancestral traits of bryophytes (Puttick *et al*., 2018; Harris *et al*., 2022). To reconstruct the ancestral chromatin landscape of bryophytes, we investigated DNA methylation and a set of five histone H3 PTMs in the model hornwort *Anthoceros agrestis* (Frangedakis *et al*., 2023) and compared it with *P*. *patens* and *M*. *polymorpha*. We conclude that the chromatin organization of bryophytes is distinct from that described in other groups of eukaryotes. It is a patchwork of transposable elements (TEs) and genes forming small units of euchromatin and heterochromatin without segregation of large heterochromatin domains around centromeres.

## Materials and Methods

### Plant materials

*Anthoceros agrestis* (Paton) Damsholt strain Oxford was cultured on 0.5 Gamborg's B5 medium solidified with 1% agar under continuous white light at 22°C.

### Chromosomal assembly of *A*. *agrestis* Oxford strain genome

DNA extracted from axenic cultures was sequenced using Oxford Nanopore MinION (ONT) and a total of 11.8 Gb of long‐read sequences were obtained. A draft assembly was constructed from the longest ONT reads that yield 50× coverage using Flye 2.9 (Kolmogorov *et al*., 2019). The assembly was error‐corrected with Pilon 1.24 (‘java ‐Xmx100G ‐jar pilon‐1.24.jar ‐‐genome ‐‐frags –nanopore’; Walker *et al*., 2014) using Illumina genomic data from Li *et al*. (2020). Hi‐C data were generated and used to scaffold the assembly by Phase Genomics (Seattle, WA, USA). Gaps between contigs in the scaffolded assembly were filled by TGS‐GapCloser (‘tgsgapcloser –scaff ‐‐reads ‐‐ouput ‐‐ngs ‐‐pilon ‐‐samtools –java’; Xu *et al*., 2020) using ONT DNA sequences. One 7.4 Mb contig with an unusually high GC content (72%), very low coverage in the Illumina dataset (< 1×), and high identity Blast hits to *Actinobacteria* was removed as a likely contaminant. A custom repetitive element library was constructed with EDTA 2.0 (EDTA.pl ‐‐sensitive 1 ‐‐anno 1 ‐‐evaluate 1 ‐t 12 ‐‐genome –repeatmasker –cds; Ou *et al*., 2019) and used to mask repeats throughout the genome. Braker2 v.2.1.5 (‘braker.pl ‐‐genome ‐‐bam ‐‐prot_seq ‐‐prg=gth ‐‐gth2traingenes ‐‐verbosity 3 ‐‐cores 12 ‐‐nocleanup ‐‐softmasking ‐‐GENEMARK_PATH ‐‐AUGUSTUS_CONFIG_PATH ‐‐AUGUSTUS_BIN_PATH ‐‐AUGUSTUS_SCRIPTS_PATH’; Brůna *et al*., 2021) was used with a combination of RNA‐seq data from (Li *et al*., 2020) mapped to the repeat‐masked genome with HISAT2 (Kim *et al*., 2019), and genes previously predicted in hornworts (Li *et al*., 2020; Zhang *et al*., 2020). Genome and annotation completeness were assessed by Busco v.5 with viridiplantae_odb10 dataset (Manni *et al*., 2021), and LTR assembly index (Ou *et al*., 2018) with default parameters.

### Annotation of TEs in *A*. *agrestis*

Transposable elements of *A*. *agrestis* were annotated using EDTA 2.0.1 (‘‐‐species others ‐‐sensitive 1 ‐‐threads 15 ‐‐anno 1 ‐‐force 1’; Ou *et al*., 2019), which incorporates several tools, including LTRharvest, LTR_FINDER, LTR_retriever, Generic Repeat Finder, TIR‐Learner, MITE‐Hunter, HelitronScanner, and RepeatMasker. All tools were adjusted to EDTA with proper filters and parameters. Final, nonredundant TE libraries were produced by removing nested insertions and protein coding genes with EDTA using customized scripts.

### Chromatin profiling of *A*. *agrestis* using ChIP‐seq

ChIP experiments were performed using a previously described protocol with some modifications (Yelagandula *et al*., 2017). Four‐week‐old gametophyte tissue of *A*. *agrestis* were collected and cross‐linked using 1% paraformaldehyde in 1× PBS under vacuum on ice for 10 min. The cross‐linking reaction was quenched by adding 2 M glycine under vacuum on ice for 10 min. Excess solution was removed from cross‐linked tissue by blotting with paper towels. Cross‐linked tissue was then snap frozen in liquid nitrogen and ground to a fine powder using mortar and pestle. The powder was transferred into a 50‐ml plastic tube and suspended in 40 ml of MP1 buffer (10 mM MES‐KOH buffer pH 5.3, 10 mM NaCl, 10 mM KCl, 0.4 M sucrose, 2% (w/v) PVP‐10, 10 mM MgCl_2_, 10 mM 2‐mercaptoethanol, 6 mM EGTA, 1× cOmplete protease inhibitor cocktail). Suspended samples were then filtered twice through one layer of Miracloth, once through a 40 μm nylon mesh, and twice through a 10 μm nylon mesh. Filtered samples were centrifuged at 3000 × ***g*** at 4°C for 10 min, and the supernatant was discarded. The pellet was washed using 15 ml of MP2 buffer (10 mM MES‐KOH buffer pH 5.3, 10 mM NaCl, 10 mM KCl, 0.25 M sucrose, 10 mM MgCl_2_, 10 mM 2‐mercaptoethanol, 0.2% Triton‐X 100, 1× cOmplete protease inhibitor cocktail) three times. The final pellet was then resuspended in 5 ml of MP3 buffer (10 mM MES‐KOH buffer pH 5.3, 10 mM NaCl, 10 mM KCl, 1.7 M sucrose, 2 mM MgCl_2_, 10 mM 2‐mercaptoethanol, 1× cOmplete protease inhibitor cocktail) and centrifuged at 16 000 × ***g*** at 4°C for 1 h. After centrifugation, the supernatant was discarded, and the pellet was resuspended in 900 μl of covaris buffer (0.1% SDS, 1 mM EDTA, 10 mM Tris–HCl pH 8.0, 1× cOmplete protease inhibitor cocktail). The resuspended pellet containing the chromatin fraction was fragmented using a Covaris E220 High‐Performance Focused Ultrasonicator for 15 min at 4°C (duty factor, 5.0; peak incident power, 140.0; cycles per burst, 200) in a 1‐ml Covaris milliTUBE. Sheared chromatin was centrifuged at 20 000 × ***g*** at 4°C for 10 min, and the supernatant was transferred into a new 5‐ml tube and diluted by adding 2.7 ml of ChIP dilution buffer. Diluted chromatin was cleaned by incubating with proteinA/G beads (Thermo Fisher Scientific, Waltham, MA, USA) at 20 rpm spinning at 4°C for 1 h. Beads were removed by magnetic racks and precleared chromatin was separated into six tubes and incubated with 1 μg of specific antibodies for histone modifications (Supporting Information Table S1) at 20 rpm spinning at 4°C overnight. Chromatin bound by antibodies was collected by incubating with protein A/G beads for 3 h. The beads were collected by magnetic racks and washed twice with a low salt wash buffer (20 mM Tris–HCl pH 8.0, 150 mM NaCl, 2 mM EDTA, 1% Triton X‐100 and 0.1% SDS), once with a high salt wash buffer (20 mM Tris–HCl pH 8.0, 500 mM NaCl, 2 mM EDTA, 1% Triton X‐100 and 0.1% SDS), once with a LiCl wash buffer (10 mM Tris–HCl pH 8.0, 1 mM EDTA, 0.25 M LiCl, 1% IGEPAL CA‐630 and 0.1% sodium deoxycholate), and twice with a TE buffer (10 mM Tris–HCl pH 8.0 and 1 mM EDTA). Immunoprecipitated DNA was eluted using 500 μl elution buffer (1% SDS and 0.1 M NaHCO_3_) at 65°C for 15 min. To reverse cross‐link, eluted DNA was mixed with 51 μl of reverse cross‐link buffer (40 mM Tris–HCl pH 8.0, 0.2 M NaCl, 10 mM EDTA, 0.04 mg ml^−1^ proteinase K; Thermo Fisher Scientific), and incubated at 45°C for 3 h and then at 65°C for 16 h. After cross‐link reversal, DNA was treated with 10 μg of RNase A (Thermo Fisher Scientific), incubated at room temperature for 30 min and purified using the MinElute PCR purification kit (Qiagen). ChIP‐seq library was generated from ChIPed DNAs using Ovation® Ultralow Library Systems V2 (Tecan, Männedorf, Switzerland). The ChIP‐seq libraries were sequenced on illumina Hiseq v4 to generate 50 bp single end reads.

### ChIP‐seq data analyses

The bam files of ChIP‐seq reads were sorted with SAMtools v.1.9 (Li *et al*., 2009) and converted to fastq format using the bamtofastq function of BEDTools v.2.27.1 (Quinlan & Hall, 2010), trimmed with Cutadapt v.1.18 (Martin, 2011) and aligned to the *A*. *agrestis* Oxford strain genome assembled in this study using Bowtie2 v.2.3.4.2 (Langmead & Salzberg, 2012). Resulting bam files were sorted and indexed with SAMtools v.1.9. Reads with MAPQ < 10 were removed with SAMtools v.1.9 and duplicates were removed with Picard v.2.18.27 (http://broadinstitute.github.io/picard/). Pearson's correlation matrices were generated using multiBamSummary and plotCorrelation functions in deepTools v.3.3.1 (Ramírez *et al*., 2016). Deduplicated reads from two biological replicates were merged. The read coverage of each chromatin mark was normalized against the read coverage of H3 in 10 bp windows with the bamCompare function in deepTools v.3.3.1, generating bigwig files. Broad peaks of each chromatin mark were called by using macs2 v.2.2.5 with default settings (Zhang *et al*., 2008). Overlaps between genomic features and each chromatin mark were calculated by using the intersect function of BEDTools v.2.27.1. The ratio of each genomic feature was calculated by dividing the total length of overlaps by the total length of each chromatin mark.

### Clustering analysis of ChIP‐seq data

*K*‐means clustering of chromatin marks was performed using deepTools v.3.3.1. Matrices were computed using the computeMatrix function of deepTools v.3.3.1 with the reference‐point subcommand or scale‐regions subcommand for protein coding genes (PCGs) or TEs, respectively, using bigwig files as the input. These matrices were imported into R v.4.2.0 using profileplyr package, and within‐groups sum of squares were calculated and plotted against number of clusters to estimate optimal numbers of clusters. Heatmaps of matrices were plotted with plotHeatmap with *k*‐means clustering (*k* = 5 for PCGs and *k* = 8 for TEs). Overlaps between PCG annotations and TE annotations were calculated using the intersect function of BEDTools v.2.27.1. Protein coding genes are considered as overlapped by TEs when > 25% of the regions of each PCG are overlapped by each TE and vice versa. Numbers of PCGs overlapped by TEs and TEs overlapped by PCGs per cluster were plotted using the ggplot2 package in R (Wickham, 2016). Genome mappability of the *A*. *agrestis* Oxford strain genome assembled in this study was calculated by using GenMap (Pockrandt *et al*., 2020) with options ‐K 50 and ‐E 0. The output bedgraph file was converted to a bigwig file by using bedGraphToBigWig v.385 (Kent *et al*., 2010). Average mappability of each PCG and TE was calculated using bigWigAverageOverBed (Kent *et al*., 2010) and plotted by using the ggplot2 package in R (Wickham, 2016).

To assign the closest features of each PCG or TE, all PCGs and TEs overlapped by TEs and PCGs respectively, were removed by using the intersect function of BEDTools v.2.27.1. Then, the nearest genomic feature to each PCG was assigned by comparing the PCG annotation with both PCG and TE annotations using closest function of BEDTools v.2.27.1 with options ‐io, ‐mdb all, ‐D a, ‐t first and either ‐id or ‐iu. The nearest genomic feature to each TE was calculated similarly. These data were plotted by using the ggplot2 package in R (Wickham, 2016).

### Gene expression analysis of *A*. *agrestis*

Gene expression data from Li *et al*. (2020) were downloaded from the SRA (PRJNA574453) or ENA (PRJEB34743) and processed with RSEM v.1.2.31 (Li & Dewey, 2011) and STAR v.2.5.2a (Dobin *et al*., 2013). Transcript Per Million (TPM) values were averaged from three biological replicates from each condition and used for further analyses. The association of each peak over PCGs was calculated using the intersect function of BEDtools v.2.27.1. Protein coding genes were considered as overlapped by each chromatin mark when > 50% of the regions of each PCG were overlapped by each peak. Average expression levels per chromatin peak were plotted using the ggplot2 package in R (Wickham, 2016). Heatmaps of expression levels of PCGs in cluster 1–4 over various ages of gametophyte and sporophyte tissues were plotted using the pheatmap function in R (Wickham, 2016).

### Genome wide profiling of 5 mC in *A*. *agrestis*

Genomic DNA was extracted from 100 mg of 4‐wk‐old gametophyte tissue of *A*. *agrestis* using Nucleon PhytoPure (cytiva). Sequencing libraries for genome wide DNA methylation profiles were generated from 200 ng of genomic DNA using NEBNext® Enzymatic Methyl‐seq Kit (New England Biolabs, Ipswich, MA, USA). Libraries from two biological replicates were sequenced on an Illumina NextSeq 2000 to generate 100 bp paired end reads.

### EM‐seq data analysis

The bam files of two biological replicates of EM‐seq reads were merged and sorted with SAMtools v.1.9 and converted to fastq format using bamtofastq function of BEDTools v.2.27.1, then trimmed with Trim Galore (https://github.com/FelixKrueger/TrimGalore). A bisulfite‐converted reference genome was prepared from *A*. *agrestis* Oxford strain genome sequence using Bismark v.0.22.2 (Krueger & Andrews, 2011). Trimmed reads were mapped to the bisulfite genome using the Bowtie2 option with default parameters of Bismark v.0.22.2. Duplicates were removed using the deduplicate function in Bismark v.0.22.2. Cytosine methylation reports were created from deduplicated reads using the bismark_methylation_extractor function in Bismark v.0.22.2. Each cytosine which is covered by at least 10 reads was used for further analyses. The methylation ratio of each cytosine was calculated and summarized to a bed file. These bed files were converted to bigwig files using bedGraphToBigWig (Kent *et al*., 2010) and used as inputs for the computMatrix function in deepTools v.3.3.1. Aggregate profile plots of matrices were plotted with the plotProfile function in deepTools v.3.3.1.

### Hi‐C contact map construction

Hi‐C reads were mapped to the *A*. *agrestis* genome with Juicer by default parameters and visualized using Juicebox (Durand *et al*., 2016). Finally, we obtained DNA contact signal of six pseudochromosomes. This visualization provides insight into the spatial organization of the genome by showing the frequency and intensity of physical interactions between different regions.

### Identification of TAD and A/B compartment

HiCExplorer v.3.3 (Ramírez *et al*., 2018) was used for the identification of A/B compartment and topologically associating domain (TAD) boundaries. Clean data were initially mapped to the *A*. *agrestis* genome using bwa mem with parameters ‘‐E50 ‐L0’. Subsequently, dangling end reads, same fragment reads, self‐circles reads, and self‐ligation reads were removed. Raw Hi‐C matrices were generated at resolutions of 20, 40, 100, and 200 kb in h5 format using hicBuildMatrix (‘‐‐binSize 10000, ‐‐restrictionSequence GATC, ‐‐danglingSequence GATC’) and hicMergeMatrixBins (‘‐numBins 2,4,10,20’). To diagnose and correct matrix resolution, the KR method was applied with hicCorrectMatrix (‘‐‐filterThreshold ‐2 2 ‐‐perchr ‐‐sequencedCountCutoff 0.2 ‐‐iterNum 1500 –correctionMethod KR’). Topologically associating domain and TAD boundaries were then identified using hicFindTADs (‘‐‐correctForMultipleTesting fdr ‐‐delta 0.01’), with TAD separation scores calculated at 40 kb resolution using default parameters. Finally, compartmentalization was performed using hicPCA (‘–extraTrack gene.bed’), with compartment A/B assignment indicated by PC1 values from the analysis on correlation maps with the lieberman method performed by hicTransform (‘‐‐method obs_exp_lieberman’). Positive and negative values of the first principle components were plotted to indicate high gene density (compartment A) and low gene density (compartment B), respectively.

### Density profile of different genomic features

The density profile was calculated using BEDtools v.2.27 (Quinlan & Hall, 2010) as the total number of peaks or DNA methylation sites in each window, divided by window length (40 kb). Figures were generated using ggplot2.

### Functional annotation of PCGs

We used BlastP v.2.2.26 (Johnson *et al*., 2008) to identify homologous genes within the *A*. *agrestis* genome that correspond to the *M*. *polymorpha* sex chromosome genes with parameter ‘‐evalue 1e‐10 ‐max_target_seqs 1 ‐outfmt 6’. In order to obtain functional annotations of PCGs in the cluster P2, we performed Interproscan v.5.57‐90.0 (Quevillon *et al*., 2005) and eggNOG‐mapper v.2 (Cantalapiedra *et al*., 2021) analyses to identify their potential functions based on homology. Additionally, we carried out Gene Ontology (GO) enrichment analysis on each PCG cluster using ClusterProfile v.3.17 (Wu *et al*., 2021).

## Results

### Chromatin profiling of *A. agrestis*

**Fig. 1:**
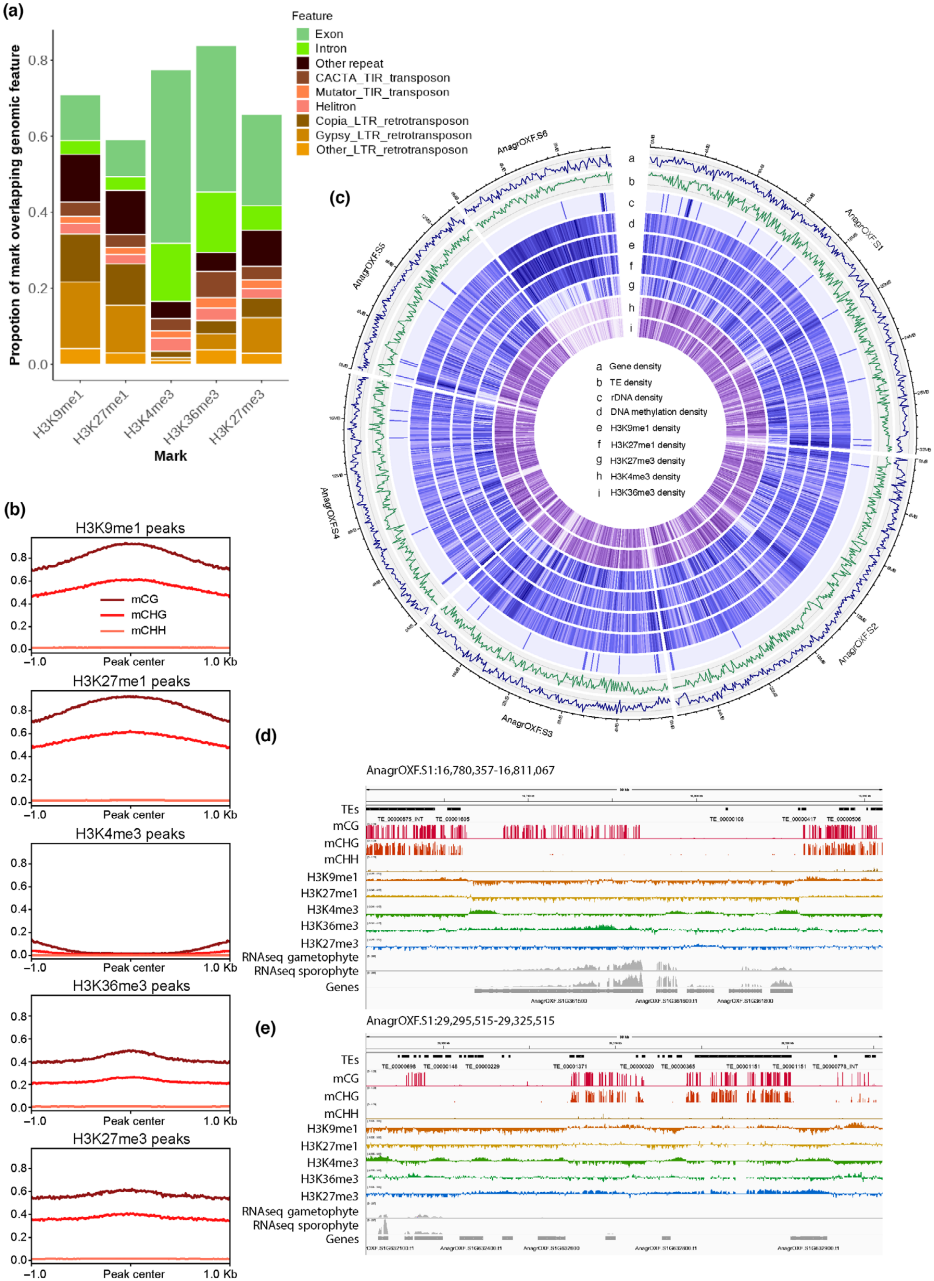
Association of chromatin marks with genomic features. (a) Distribution of histone posttranslational modifications (PTMs) over genomic features. The total length of PTMs overlapping specified genomic features was divided by the total length of PTM peaks to determine each proportion. Features that cover < 0.5% of PTM peaks are not shown. H3K9me1, H3K27me1 are expected as PTMs of constitutive heterochromatin and H3K27me3 is a PTM of facultative heterochromatin with a distribution contrasting with the active PTMs H3K4me3 and H3K36me3. (b) Profile plot of CG, CHG, and CHH methylation levels over PTM peaks. Sequences 1 kb upstream and downstream of the peak center are included. Average methylation over 10 bp bins is plotted. (c) Genomic features of the *Anthoceros agrestis* genome. The Circos plot illustrates the genomic features of the *A*. *agrestis* genome using rings to display different information with a window size of 100 kb. The rings represent the following features: (a) gene density, (b) transposable element (TE) density, (c) ribosomal DNA (rDNA) density, (d) DNA methylation density, (e) H3K9me1 peak density, (f) H3K27me1 peak density, (g) H3K27me3 peak density, (h) H3K27me3 peak density, (i) H3K36me3 peak density. (d, e) Integrative Genomics Viewer (IGV) browser screenshot demonstrating vicinity of TEs covered by H3K9me1 and expressed genes (d) and TEs covered by H3K27me3 (e). The regions shown are 30 kb in length from the scaffold AnagrOXF.S1. Posttranslational modifications tracks are bigwig files scaled by H3 coverage in 10‐bp windows. DNA methylation tracks are bigwig files showing methylation levels of each cytosine site covered by at least 10 reads. ‘TEs’ and ‘Genes’ tracks are annotation files for TEs and genes, respectively. ‘RNA‐seq’ tracks are bigwigs of mapped RNA‐seq reads from gametophyte tissue and sporophyte tissue (Li *et al*., 2020). Scales are noted in square brackets in each track.

We used Enzymatic Methyl‐seq (Vaisvila *et al*., 2021) to obtain a genome wide profile of 5‐methyl cytosines from 4‐wk‐old vegetative tissue after transfer to new growth media of *A*. *agrestis*. Chromatin immunoprecipitation coupled with DNA sequencing (ChIP‐seq) was applied to the same tissue to obtain genomic profiles of five histone PTMs (H3K4me3, H3K36me3, H3K9me1, H3K27me1, and H3K27me3) and H3. Although H3K9me2 is often considered as a mark for constitutive heterochromatin, we did not detect peaks of enrichment for this PTM in *A*. *agrestis*. Instead, we used H3K9me1 as a mark for constitutive heterochromatin as this PTM shows similar coverage as H3K9me2 in *M*. *polymorpha* and represents broadly constitutive heterochromatin in *A*. *thaliana*. Peaks from each replicate of all five marks exhibited a significant overlap between each other (Table S2). Furthermore, biological replicates clustered together in a Pearson's correlation matrix (Fig. S1a) and the profiles of PTMs typically considered repressive (H3K9me1, H3K27me1, and H3K27me3) or active (H3K4me3 and H3K36me3) formed two clusters of high similarity (Fig. S1a), showing the robustness of our data. In addition, we re‐annotated TEs in the *A*. *agrestis* Oxford strain and identified 88 959 TEs, including 1155 intact TEs belonging to various TE families (Dataset S1). A large majority of the annotated TEs were relatively short and, apart from MITEs, were primarily fragments of intact TEs. Peaks of H3K9me1 and H3K27me1 were associated with high levels of DNA methylation in both CG and CHG contexts and primarily associated with various types of TEs but also some PCGs (Fig. 1a,b). Peaks of H3K4me3 were enriched in PCGs and with very low levels of DNA methylation (Fig. 1a,b). Unlike these associations that are observed in other model land plants, peaks of H3K36me3 and H3K27me3 were not only present on PCGs but also on TEs with modest levels of cytosine methylation (Fig. 1a,b). While in the *A*. *agrestis* genome, many of the associations between genomic features and PTMs were identical to those reported in other model plants, this overview suggested additional associations present in hornworts.

To investigate the chromosome‐level organization of the chromatin landscape in *A*. *agrestis*, we upgraded the existing genome assembly of the *A*. *agrestis* Oxford strain (Li *et al*., 2020) by generating additional nanopore long reads as well as Hi‐C data. The contig N50 of the new assembly increased from 1.8 to 7.0 Mb. Furthermore, contigs were placed into six large scaffolds corresponding to the six chromosomes in *A*. *agrestis*, representing 98% of the total assembly with a BUSCO completeness of 92% and LTR assembly index of 19.8. Using this assembly, we plotted densities of PCGs, TEs, and PTMs to see the distribution of these features over chromosomes (Fig. 1c). Overall, genes and TEs were evenly distributed over the chromosome of *A*. *agrestis* Oxford strain as reported previously in the *A*. *agrestis* Bonn strain (Li *et al*., 2020). Corresponding to this pattern, all PTMs were distributed evenly over the chromosomes, except for chromosome 6 discussed below (Fig. 1c). We observed active PCGs covered by euchromatic marks and surrounded by TEs covered by heterochromatic marks (Fig. 1d). We also observed relatively long genomic regions that included both PCGs and TEs covered by H3K27me3 (Fig. 1e). This even distribution of PTMs, genes, and TEs observed in chromosomes one to five was comparable to the chromatin organization in *M*. *polymorpha* and *P*. *patens* (Widiez *et al*., 2014; Montgomery *et al*., 2020).

**Fig. 2:**
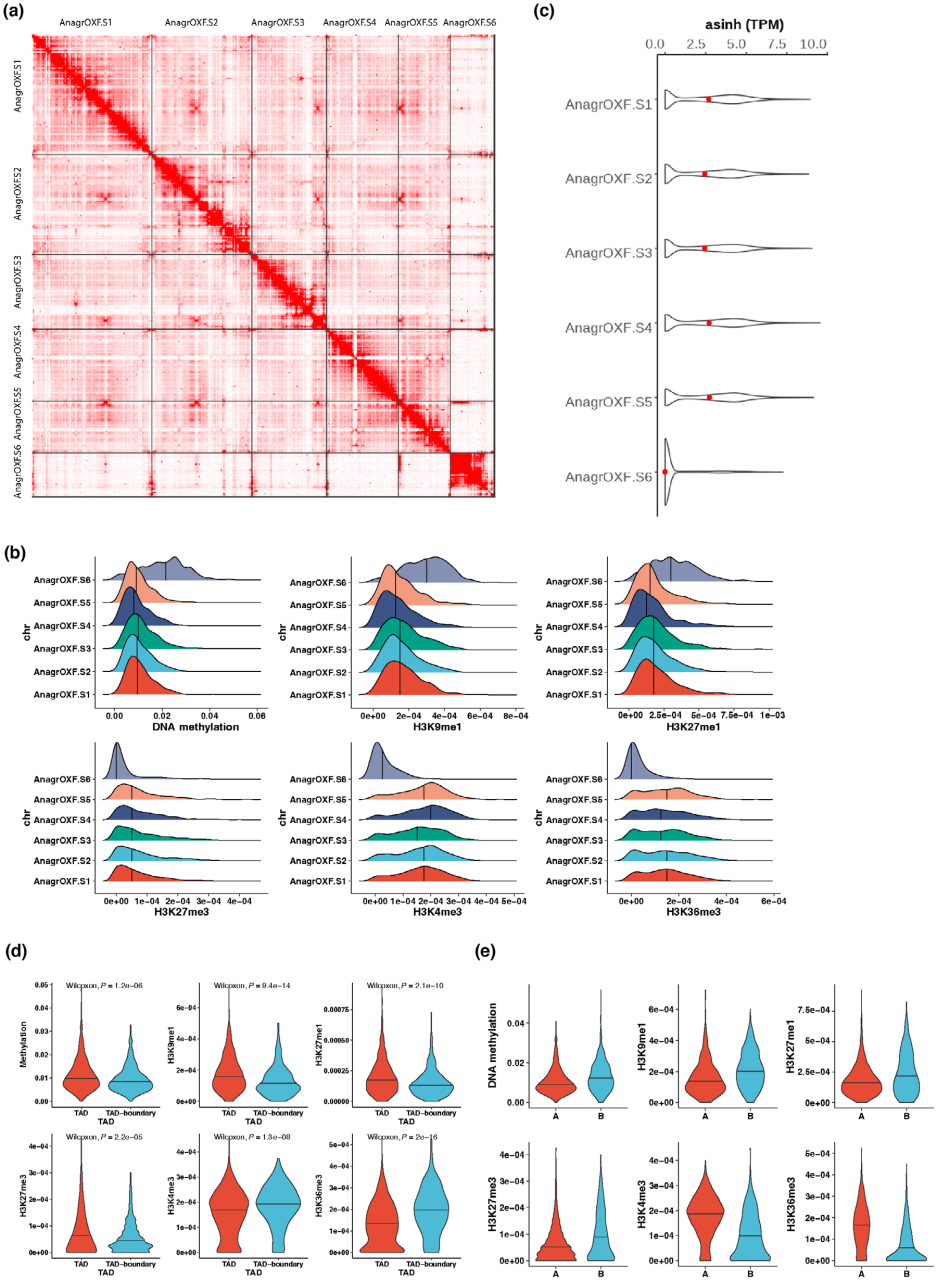
Higher order structure of *Anthoceros agrestis* chromosomes. (a) Hi‐C contact heatmap of chromosomes. The blocks were utilized to symbolize the signal linked with the two contact positions. The color depth corresponds to the intensity of the interaction between DNA molecules. The darker shades indicate a stronger interaction between them. (b) Density plots showing distributions of epigenetic marks per chromosome. The density of DNA methylation or histone posttranslational modifications (PTMs) were calculated as the number of DNA methylation sites or numbers of peaks of histone PTMs in each 40 kb window, divided by window size (40 kb), and plotted for each chromosome. The median value for each chromosome is represented by a solid vertical line. (c) Violin plot showing expression level of protein coding genes (PCGs) per chromosome. Expression levels are indicated by Transcript per Million (TPM) values transformed by using asinh function. Width is relative to PCG density. Red dots indicate median expression values. (d) Violin plots showing density distribution of epigenetic marks in topologically associating domains (TADs) and TAD boundaries. The density of epigenetic marks was calculated as the number of histone PTM peaks or DNA methylation sites in each 40 kb window, divided by window length, and plotted for TADs and TAD boundaries. The median value is represented by a solid horizontal line. *P*‐values from the Wilcoxon test are indicated on each plot. (e) Violin plots showing density distribution of epigenetic marks in different compartments. The density of epigenetic marks calculated as above is plotted against A or B compartment. The median value is represented by a solid horizontal line.

In contrast to the even distribution of PCGs, TEs, and associated PTMs over these five chromosomes, there were fewer PCGs and more TEs on chromosome 6 and most of this chromosome was covered by DNA methylation, H3K9me1, and H3K27me1 (Figs 1c, S1b,c; Table 1). In the Hi‐C contact map, we observed that chromosome 6 has more intrachromosomal contacts and fewer interchromosomal contacts than the other chromosomes (Fig. 2a). We also calculated the densities of each PTM per chromosome and found that chromosome 6 was mostly occupied by constitutive heterochromatin with high densities of DNA methylation, H3K9me1, and H3K27me1 and low densities of H3K4me3, H3K36me3, and H3K27me3 than the other chromosomes (Fig. 2b). Corresponding to these heterochromatic characteristics, PCGs on chromosome 6 were expressed at a lower level than PCGs on the other five chromosomes (Fig. 2c). The characteristics of chromosome 6 were similar to those of the sex chromosomes in the dioicous species *M*. *polymorpha* (Montgomery *et al*., 2020). However, chromosome 6 did not show an enrichment of orthologs of genes present on *M*. *polymorpha* sex chromosomes (Dataset S2; Table 2). *Anthoceros agrestis* is monoicous, and the association of chromosome 6 with constitutive heterochromatin remains enigmatic. To explore relationships between higher order chromosomal structure and epigenetic marks, we annotated TADs and split the genome into two compartments (active A compartment and inactive B compartment) based on the Hi‐C data and calculated the density of each epigenetic mark in TADs or in A/B compartments. We observed a slight enrichment of repressive marks (DNA methylation, H3K9me1, H3K27me1, and H3K27me3) and TEs inside the TADs and a slight enrichment of active marks (H3K4me3 and H3K36me3) and PCGs at TAD boundaries (Figs 2d, S1d). The A compartments showed enrichment of active marks while repressive marks identified the B compartments (Fig. 2e). The centromeres of the chromosomes of *A*. *agrestis* were not conspicuous based on the Hi‐C map and were not marked by a strong accumulation of transposable elements (Figs 1c, 2a). These features of TAD, A/B compartments, and centromeres were similar to those described in *M*. *polymorpha* (Montgomery *et al*., 2020) and *P*. *patens* (Bi *et al*., 2023). We conclude that the overall genome organization of bryophytes shows similar features.

**Table 1:** Summary of protein coding genes (PCGs) and transposable elements (TEs) proportions per chromosome.

	Total length (Mbp)	PCG occupancy (Mbp)	PCG proportion (%)	PCG counts	TE occupancy (Mbp)	TE proportion (%)	TE counts
AnagrOXF.S1	32.3	11.8	36.5	6864	12.1	37.5	21 951
AnagrOXF.S2	27.0	9.8	36.5	5827	10.8	39.9	18 853
AnagrOXF.S3	20.0	7.0	34.9	4136	8.4	42.0	14 600
AnagrOXF.S4	19.2	7.9	40.9	4455	6.5	33.7	11 756
AnagrOXF.S5	14.1	5.6	40.0	3173	4.5	32.3	8147
AnagrOXF.S6	11.8	2.5	21.6	2170	8.1	68.8	10 960
Total	124.4	44.7	35.9	26 625	50.4	40.5	86 267

**Table 2:** Number of homologs of sex chromosome genes in *Marchantia polymorpha* per chromosome.

chr	AnagrOXF.S1	AnagrOXF.S2	AnagrOXF.S3	AnagrOXF.S4	AnagrOXF.S5	AnagrOXF.S6
All	6864	5827	4136	4455	3173	2170
sex.chr.homologs	11	12	15	5	2	3

### Association between histone PTMs and DNA methylation with protein coding genes

**Fig. 3:**
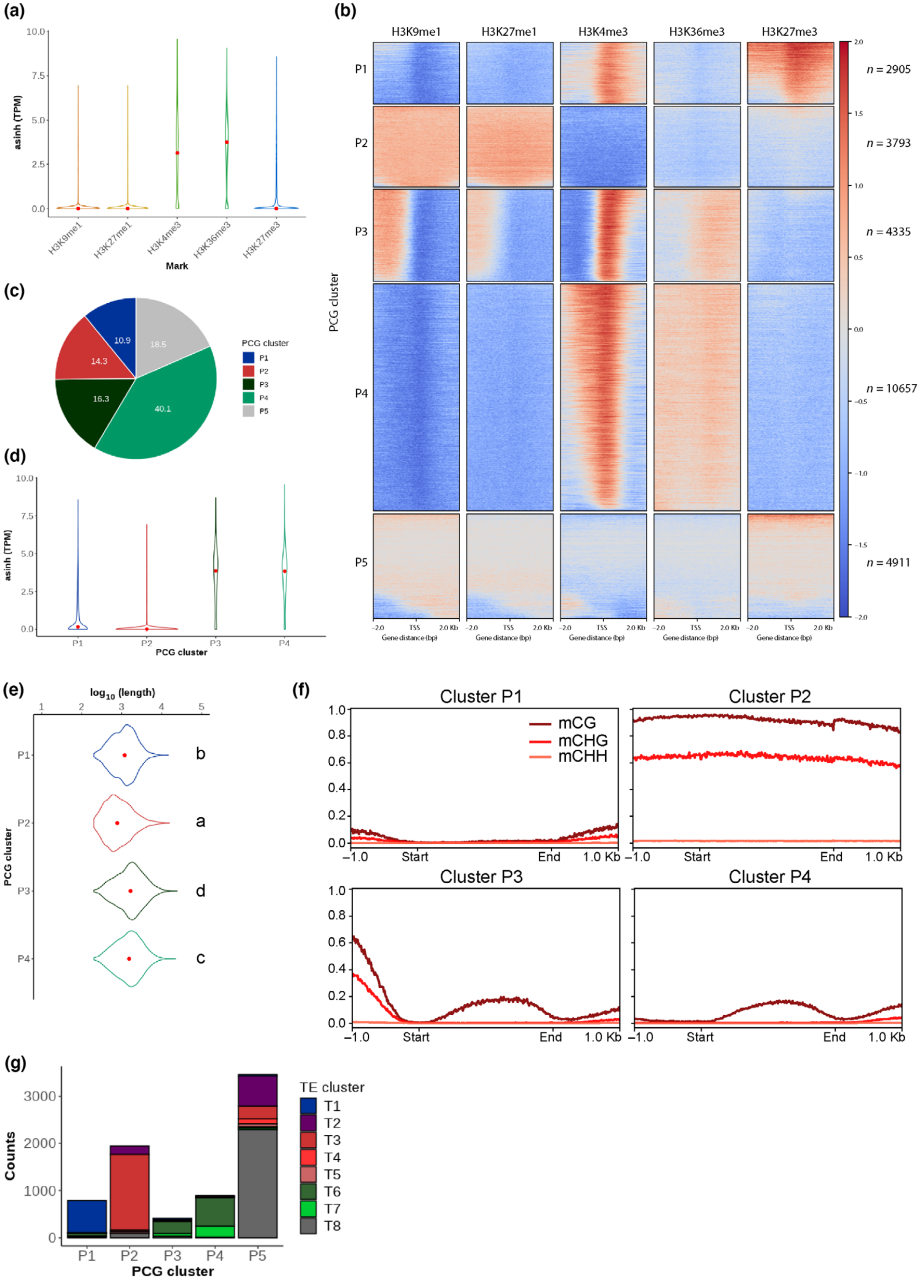
Association of chromatin marks on protein coding genes. (a) Violin plot showing expression level of protein coding genes (PCGs) associated with histone posttranslational modifications (PTMs). Expression levels are indicated by Transcript per Million (TPM) values transformed by using asinh function. Width is relative to PCG density. Red dots indicate median expression values. (b) Heatmap of *k*‐means clustering of genes based on PTMs. Prevalence of each mark (columns) based on its score normalized against H3 signals per 10 bp bins. Sequences 2 kb upstream and downstream of the start codon are included. Red stands for enrichment and blue for depletion. Each row corresponds to one gene, with multiple genes grouped into blocks that have been defined as clusters P1 through P5. (c) Pie chart showing proportions of PCG clusters in all PCGs. (d) Violin plot showing expression level of PCGs per PCG cluster. Expression levels are indicated by TPM values transformed by using asinh function. Width is relative to the density of PCGs. Red dots indicate median expression values. (e) Violin plot showing length of PCG per PCG cluster. The width is relative to the density of PCGs. Red dots indicate median values. Clusters not sharing the same letter are significantly different (Tukey–kramer test, *P* < 0.05). (f) Profile plot of CG, CHG, and CHH methylation levels over PCGs per PCG cluster. Gene body of each PCG is scaled to 2 kb and sequences 1 kb upstream and downstream are included. Average methylation over 10 bp bins is plotted. (g) Stacked bar chart showing numbers of PCGs overlapped by transposable elements (TEs) at least 25% of their length per PCG cluster. Different colors indicate TE clusters defined in Fig. (4a).

We explored preferential associations between PTMs and the transcriptional status of PCGs based on their expression levels in vegetative tissue (thallus) from publicly available data (Li *et al*., 2020). H3K36me3 and H3K4me3 were strongly associated with expressed PCGs (Fig. 3a), while H3K9me1, H3K27me1, and H3K27me3 were associated with repressed PCGs (Fig. 3a). To discover whether there were relationships between chromatin profiles and PCGs in *A*. *agrestis*, we performed unsupervised *k*‐means clustering of chromatin profiles over all PCGs (*n* = 26 601). By calculating within‐cluster sum of square (Fig. S2a), we defined five major clusters of PCGs (named cluster P1–P5), which exhibited different chromatin environments (Dataset S3; Fig. 3b). Cluster P5, comprising 18.5% of all PCGs (Fig. 3c), was only weakly enriched for any marks examined, likely due to the difficulty of mapping reads to these predicted PCGs (Fig. S2c), and we did not consider this cluster further. We calculated the average expression level in the other clusters. Clusters P3 and P4 comprised 16.3%, and 40.1% of all PCGs, respectively, and accounted for expressed PCGs (Fig. 3b–d). These PCGs were longer than repressed PCGs (Fig. 3e). In these two clusters, H3K36me3 was enriched over gene bodies and H3K4me3 was enriched at transcription start sites (TSS), as described in other land plants (Figs 3b, S2e). Enrichment of H3K9me1, H3K27me1, and DNA methylation in the promoter region of PCGs differentiated cluster P3 from cluster P4 (Fig. 3b,f). Despite the association of these marks with heterochromatin, there was no difference in expression levels of PCGs in these two clusters (Fig. 3d). In these two clusters, gene bodies showed low levels of CG methylation and no cytosine methylation in non‐CG contexts (Fig. 3f), similar to what has been described in *P*. *patens* (Zemach *et al*., 2010) and *M*. *polymorpha* (Takuno *et al*., 2016; Ikeda *et al*., 2018). We conclude that P3 and P4 comprise euchromatin and expressed PCGs that are devoid of gene body methylation in bryophytes.

Nonexpressed PCGs formed clusters P1 and P2 (Fig. 3b–d). Cluster P1 contained 10.9% of all PCGs and was characterized by enrichment of H3K27me3 and H3K4me3 and depletion of cytosine methylation in all contexts, and was similar to cluster 1 of *M*. *polymorpha* PCGs (Montgomery *et al*., 2020). Cluster P2 contained 14.3% of all PCGs with enrichment of H3K9me1, H3K27me1, and a high level of cytosine methylation in CG and CHG contexts. No comparable chromatin state was observed over PCGs in *M*. *polymorpha* (Montgomery *et al*., 2020). Protein coding genes in cluster P2 were shorter than those in other clusters (Fig. 3e). About 60% of PCGs from cluster P2 overlapped with annotated TEs (Fig. 3g), suggesting that these PCGs are coding regions of intact TEs or PCGs that have co‐opted coding regions of TEs.

To test whether repressed PCGs from clusters P1 and P2 were expressed in nonvegetative tissue, we explored other publicly available transcriptome datasets (Li *et al*., 2020). While PCGs in clusters P3 and P4 were ubiquitously expressed in both gametophyte and sporophyte, we found that 15% of PCGs in cluster P1 were repressed in gametophytes but expressed in sporophytes, suggesting H3K27me3 functions as a facultative heterochromatic mark to regulate expression of genes regulating the distinct developmental programs of the haploid and diploid phases of the life cycle of *A*. *agrestis* (Fig. S3a,c,d). To further gain insight into the function of PCGs covered by H3K27me3, we performed GO term enrichment analysis on PCGs in cluster P1 and found that these PCGs are enriched in response genes (Dataset S4). Similar association of PRC2 repression on response genes was found in *M*. *polymorpha* (Hisanaga *et al*., 2023). Surprisingly, 9.3% of PCGs in cluster P2 were repressed in gametophytes but expressed in sporophyte stage, suggesting that H3K9me1 and/or H3K27me1 also behave as facultative heterochromatic marks in *A*. *agrestis* (Fig. S3b). These PCGs did not overlap with annotated TEs and encoded proteins such as the GDSL lipase involved in the production of cuticle (Shen *et al*., 2022), transporters, and pectin lyase, which altogether might have physiological functions during the sporophyte stage and its adaptation to a terrestrial lifestyle (Dataset S5).

We conclude that the patterns of enrichment of H3K4me3 and H3K36me3 overexpressed genes in euchromatin are broadly conserved in land plants, while in bryophytes, facultative heterochromatin associates H3K27me3 with modifications H3K9me1 and H3K27me1 that are usually distinctive of constitutive heterochromatin and TEs. In the lineage of land plants, this form of heterochromatin is unique to bryophytes and represses gene expression in a context‐dependent manner.

### Association between histone PTMs and DNA methylation on TEs

**Fig. 4:**
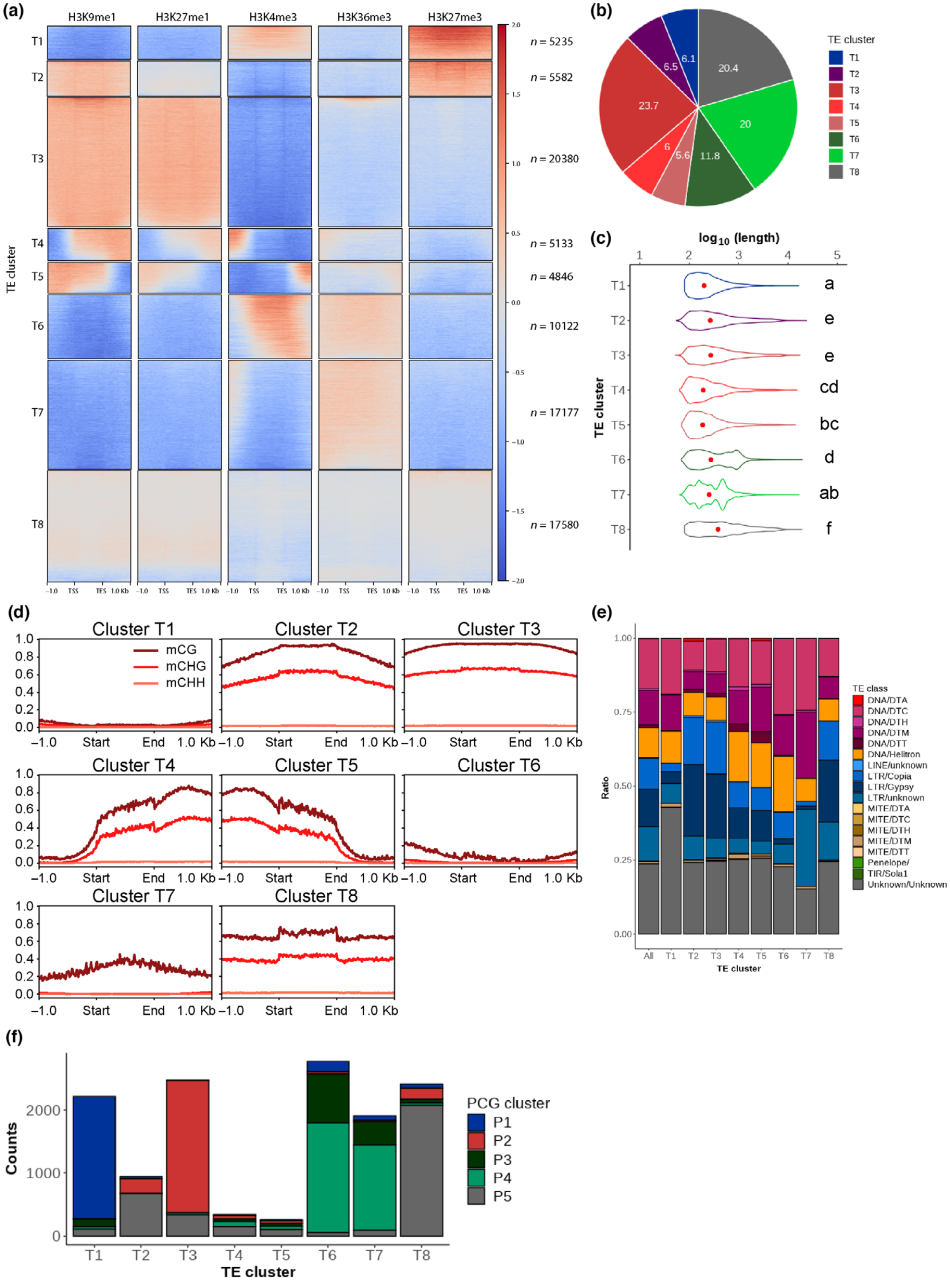
Association of chromatin marks on transposable elements (TE). (a) Heatmap of *k*‐means clustering of TEs based on histone posttranslational modifications (PTMs). Prevalence of each PTM (columns) based on its score normalized against H3 signals per 10 bp bins. Each TE annotation is scaled to 1 kb and sequences 1 kb upstream and downstream are included. Red color stands for enrichment and blue for depletion. Each row corresponds to one TE, with multiple TEs grouped into blocks that have been defined as clusters T1 through T8. (b) Pie chart showing proportions of TE clusters in all TEs. (c) Violin plot showing length of TEs per TE cluster. Width is relative to the density of TEs. Red dots indicate median values. Clusters not sharing the same letter are significantly different (Tukey–kramer test, *P* < 0.05). (d) Profile plot of CG, CHG, and CHH methylation levels over TEs per TE cluster. Each TE annotation is scaled to 1 kb and sequences 1 kb upstream and downstream are included. Average methylation over 10 bp bins is plotted. (e) Stacked bar chart indicating proportions of TE families in each TE cluster (T1–T8) in comparison with TE family proportion in the entire genome (All). (f) Stacked bar chart showing numbers of TEs overlapped by protein coding genes (PCGs) at least 25% of their length per TE cluster. Different colors indicate PCG clusters defined in Fig. (3b).

To address the relationships between PTMs and TEs, we performed *k*‐means clustering of histone H3 PTMs over TEs. We defined eight major clusters of TEs showing different chromatin environments (clusters T1–T8 in Dataset S6; Figs 4a, S2b). Cluster T8, containing 20.4% of all TEs (Fig. 4b), was weakly enriched for all marks examined, likely due to the difficulty of mapping (Fig. S2d). Compared with the other clusters, cluster T8 contained longer TEs, with high levels of DNA methylation (Fig. 4d) and half of them were retrotransposons (Fig. 4e). These TEs showed lower mappability suggesting that this cluster would consist of TEs with high copy numbers. Clusters T3, T4, and T5 contained 23.7%, 6%, and 5.6% of all TEs, respectively (Fig. 4b). These TEs were covered with H3K9me1, H3K27me1, and CG and CHG methylation (Fig. 4a,d). Cluster T3 contained longer TEs compared to those in clusters T4 and T5 (Fig. 4c) and were more enriched in LTR families (Fig. 4e). Clusters T4 and T5 were enriched for H3K4me3 in the upstream and downstream regions, respectively, and contained TEs shorter than TEs in cluster T3. These two clusters were enriched in DNA transposons (Fig. 4e). Hence, typical heterochromatin occupied TEs from clusters T3, T4, T5, and T8 representing slightly above half of all TEs of *A*. *agrestis*.

By contrast, chromatin states distinct from the typical constitutive heterochromatin were observed on TEs from clusters T1, T2, T6, and T7. Clusters T1 and T2 comprised 6.1% and 6.5% of all TEs, respectively (Fig. 4b). Transposable elements from these clusters were associated with H3K27me3 and either H3K4me3 (T1) or H3K9me1 and DNA methylation (T2; Fig. 4a,d). Cluster T1 contained shorter TEs and was enriched in unclassified TEs, while cluster T2 contained longer TEs and was enriched in LTR TEs (Fig. 4c,e). Clusters T6 and T7 comprised 11.6% and 20% of all TEs and were covered with euchromatic marks (H3K36me3 and H3K4me3) and low levels of CG and CHG methylation (Fig. 4a,b,d). These TEs were relatively short and enriched among DNA transposons (Fig. 4c,e). One‐fourth of T6 and 12% of T7 overlapped with PCGs (Fig. 4f), suggesting that parts of them were located inside PCGs. In conclusion, a relatively small fraction of TEs is covered by marks of constitutive heterochromatin (H3K9me1 and H3K27me1) and is associated with high levels of DNA methylation. These TEs are primarily LTR retrotransposons (Fig. S4). We propose that only a small fraction of TEs covered with constitutive heterochromatin still have the potential to transpose. The large fraction of TEs associated with facultative heterochromatin or euchromatin might rather participate in the transcriptional regulation of PCGs in constitutive heterochromatin and in euchromatin.

### Positional relationship between TEs and PCGs

**Fig. 5:**
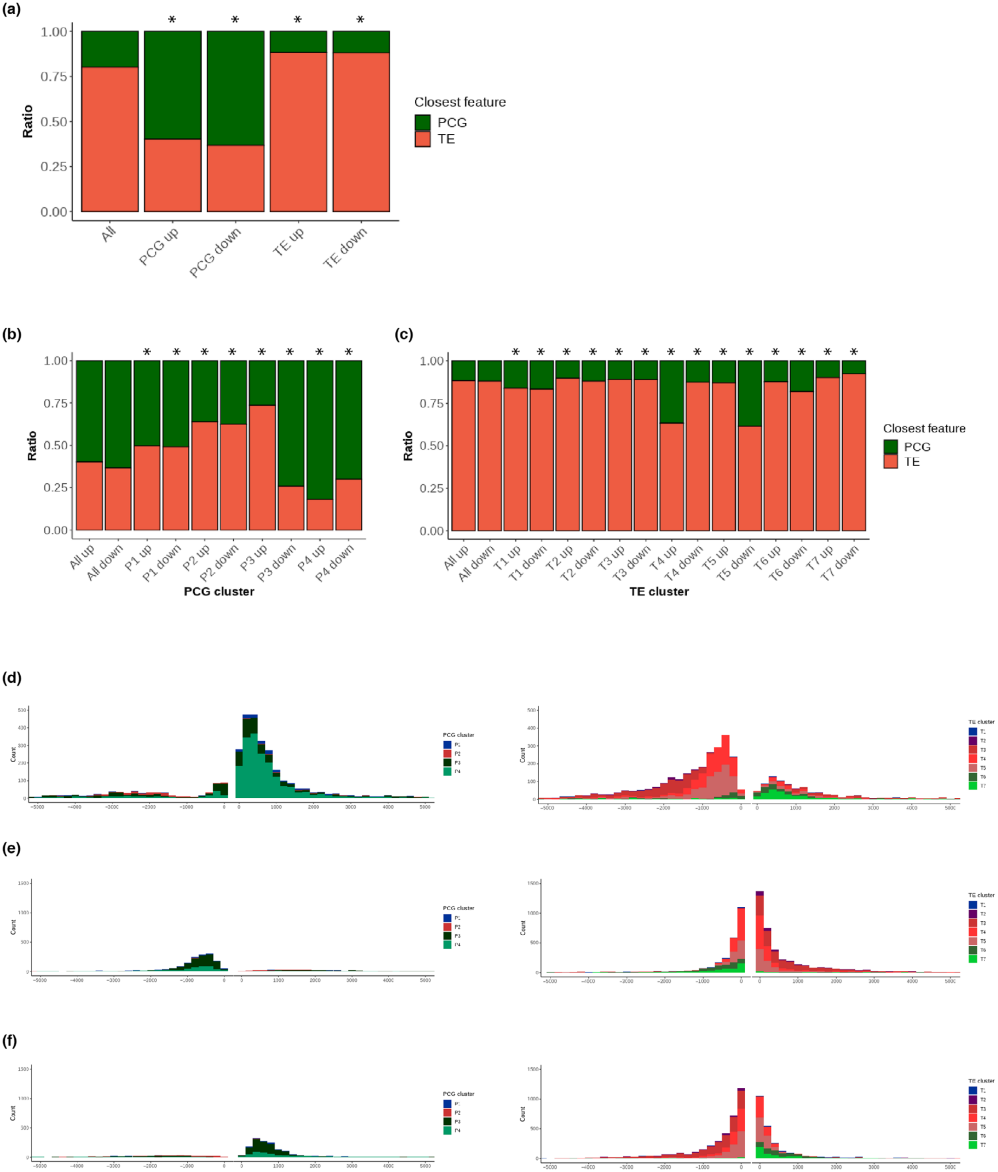
Positional relationships between protein coding genes (PCGs) and transposable elements (TEs). (a) Stacked bar chart showing the proportion of closest PCGs or TEs upstream or downstream of PCGs and TEs in comparison with the proportion in the entire genome (All). *, *P* < 2.2 × 10^−16^ (Fisher's exact test). (b, c) Stacked bar chart showing the proportion of closest PCGs or TEs upstream or downstream of PCGs (P1–P4 in (c)) and TEs (T1–T8 in (d)) per cluster in comparison with the observed proportions in the entire PCGs or TEs (All). *, *P* = 0 (permutation test). (d–f) Histograms showing distance from PCGs in P3(d) or TEs in T4 and T5 (e, f, respectively) to the closest PCGs (left) or TEs (right) per cluster.

Overall, the genome of *A*. *agrestis* comprises 20% PCGs and 80% TEs that do not segregate in large domains of constitutive heterochromatin, in contrast with the pericentromeric heterochromatin spanning few Mb in the chromosomes of the flowering plant *A*. *thaliana* (Bernatavichute *et al*., 2008). Transposable elements of *A*. *agrestis* are interspersed with PCGs at the chromosome scale. Yet, it was thus possible that PCGs or TEs form small clusters. To answer this question, we analyzed the positional relationship between PCGs and TEs. First, we removed overlapping features between the PCG annotation and TE annotation and used only nonoverlapping annotations to call the nature (PCG or TE) of the closest neighbors of each PCG and TE and plotted the PCG : TE ratio with comparison to the overall ratio of PCG/TE (1/4) (Fig. 5a). We observed that more PCGs were neighbors to PCGs and more TEs were neighbors of TEs than expected, suggesting that PCGs and TEs tended to cluster together (Fig. 5a). To test whether specific chromatin environments are associated with positional relationships between PCGs and TEs, we further compared neighboring features of each PCG and TE per cluster (Fig. 5b,c). We observed a clear difference in surrounding features of PCGs in each cluster. While PCGs covered by heterochromatic marks (P1 and P2) tended to be surrounded by more TEs than average, PCGs in the cluster P4, which are covered by euchromatic marks, were surrounded by more PCGs than average. In the cluster P3, PCGs showed more association with TEs in their upstream neighborhood and more PCGs in their downstream neighborhood. Surrounding features of TEs were more uniform among each cluster compared to PCGs except for TEs in the clusters T4 and T5, which showed a more frequent association with PCGs in the upstream and the downstream neighborhood (Fig. 5c). To test whether PCGs and surrounding TEs are more likely to share the same type of chromatin environment, we established the nature of the chromatin environment of the closest neighbors for each PCG cluster (Fig. S5a,b) or TE cluster (Fig. S5c,d). This analysis showed that PCGs (P2) and TEs (T3) covered with constitutive heterochromatin tended to be surrounded by PCGs or TEs also covered with constitutive heterochromatin (Figs S5a–d, S6a). Similarly, PCGs and TEs in euchromatin (P4, T6, and T7) were surrounded by features covered by euchromatin (Fig. S6b). This was also the case for TEs and PCGs covered with facultative heterochromatin (P1, T1, and T2; Fig. 1e) although these could be surrounded by euchromatic PCGs or TEs with constitutive heterochromatin. By contrast, the region upstream of PCGs from cluster P3 was primarily occupied by TEs from clusters T4 and T5 enriched with H3K9me1 and H3K27me1 (Figs 5d, S6c). Higher PCG ratios were found upstream of TEs from cluster T4 and downstream of TEs from cluster T5 (Fig. 5e,f). These PCGs were covered by H3K4me3 and H3K36me3 (Fig. 5e,f). We concluded that the genome of *A*. *agrestis* comprises clusters of TEs and PCGs sharing the same chromatin environment, except for P3 euchromatic PCGs with an upstream region enriched in TEs and constitutive heterochromatin.

## Discussion

**Fig. 6:**
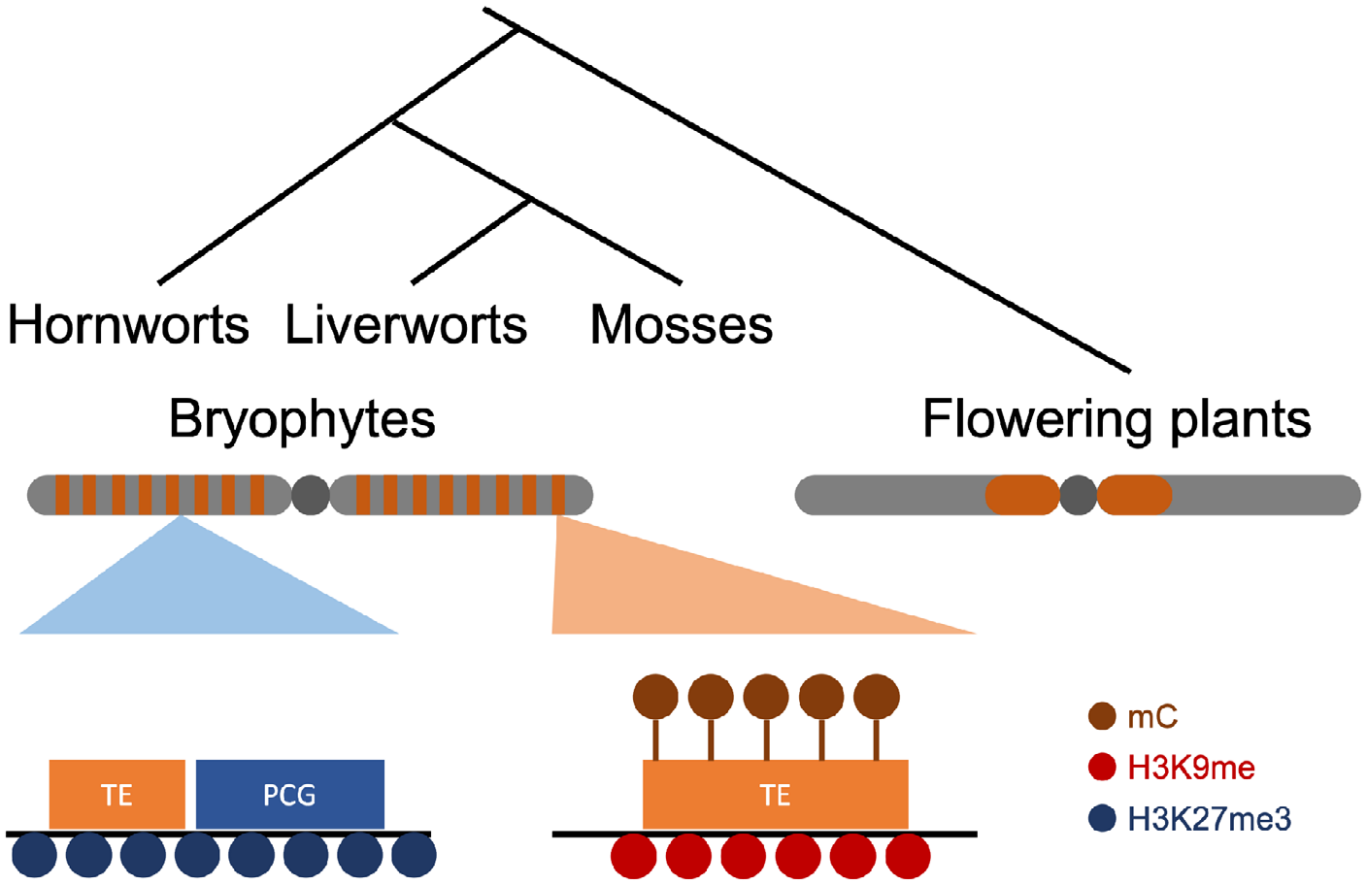
Chromatin organization in the common ancestor of bryophytes. Ancestral chromatin organization in bryophytes is inferred by chromatin synapomorphies shared by three bryophyte species. In contrast to the large pericentromeric heterochromatin observed in flowering plants (colored in orange), the constitutive heterochromatin of bryophytes forms small units and is scattered over chromosomes in bryophytes (orange bars). These constitutive heterochromatin marked by H3K9me (red circles) and DNA methylation (brown circles) primarily consist of TEs (orange box). In addition, facultative heterochromatin marked by H3K27me3 (blue circles) contains not only PCGs (blue box) but also TEs.

In flowering plants, TEs are enriched in pericentromeric heterochromatin while PCGs are enriched along chromosomal arms (Sequeira‐Mendes *et al*., 2014; Jamge & Berger, 2022). By contrast, both TEs and PCGs are evenly distributed over the entire chromosomes in bryophytes (Lang *et al*., 2018; Li *et al*., 2020; Montgomery *et al*., 2020; Zhang *et al*., 2020). Consistently euchromatin, facultative and constitutive heterochromatin are also evenly distributed over the chromosomes of *M*. *polymorpha* and *P*. *patens* (Widiez *et al*., 2014; Montgomery *et al*., 2020). In this study, we demonstrated that an even distribution of the chromatin modifications shapes the global architecture of the genome of the hornwort *A*. *agrestis* similar to *M*. *polymorpha* and *P*. *patens* (Widiez *et al*., 2014; Montgomery *et al*., 2020). The conservation of genome size and number of chromosomes among hornworts (generally 6; Villarreal & Renner, 2013) and liverworts (always 9; Bowman *et al*., 2022) also supports a general conservation of genome architecture across the bryophytes, but there might be deviations to this rule in mosses with much larger genomes (Fig. 6).

H3K9 methylation forms constitutive heterochromatin that represses the expression of TEs in many eukaryotic species (Allshire & Madhani, 2018). In addition, this mark strongly co‐occurs with H3K27me1 and DNA methylation in angiosperms (Jacob *et al*., 2009; X. Li *et al*., 2018). In all model bryophytes, the majority of TEs are also associated with H3K9me1 and H3K27me1, in agreement with the presence of SUVH4/5/6 and ATXR5/6 orthologs in bryophytes. In *M*. *polymorpha*, the ortholog of *A*. *thaliana* MET1 maintains CG methylation and is involved in silencing TEs (Ikeda *et al*., 2018). In *P*. *patens*, CHG methylation is maintained by an ortholog of *A*. *thaliana* CHROMOMETHYLASE 3 (Domb *et al*., 2020), and CHH methylation is primarily deposited by the *de novo* methyltransferase DNMT3. *Physcomitrium patens* lacks orthologs of the methyltransferases DRM1, DRM2 (Yaari *et al*., 2019), or CMT2, which are collectively responsible for CHH deposition and contribute to TE silencing in *A*. *thaliana* (Stroud *et al*., 2014; Domb *et al*., 2020). We identified orthologs of MET1 and CMT in *A*. *agrestis*, in agreement with the presence of CG and CHG methylation (Table S3). However, although DRM orthologs are present in *A*. *agrestis*, the near‐complete absence of CHH methylation suggests that the DRM ortholog is inactive in vegetative haploid tissues. DNA methylation in CHG and CG is primarily observed in TEs, suggesting the absence of gene body methylation on PCGs. Such reduction or absence of gene body methylation was also reported in *M*. *polymorpha* (Bewick *et al*., 2017) and *P*. *patens* (Domb *et al*., 2020) and is thus a common feature in bryophytes (Fig. 6).

Despite the overall conservation of chromatin environments in bryophytes, several features are specific to *A*. *agrestis*. First, a significant fraction of PCGs are surrounded by TEs forming small clusters covered by constitutive heterochromatin (H3K9me1 and H3K27me1). Strikingly, half of all TEs show very low levels of DNA methylation, suggesting that TEs play an important role outside of constitutive heterochromatin in *A*. *agrestis*. Large clusters of facultative heterochromatin comprise TEs and PCGs covered by H3K27me3. This is reminiscent of the association between TEs and H3K27me3 in *M*. *polymorpha*, further supporting a shared role of H3K27me3 in silencing TEs across a broad range of eukaryotes (Déléris *et al*., 2021; Hisanaga *et al*., 2023). On the contrary, in *A*. *agrestis*, short TE fragments covered by euchromatic marks (H3K4me3 and H3K36me3) are located near or inside PCGs. This strong association between TEs and euchromatin is not observed in *M*. *polymorpha* or *A*. *thaliana* and might result from the reduced size of the genome of *A*. *agrestis* (130 Mb and 26 000 PCGs) compared with *M*. *polymorpha* (230 Mb and 20 000 PCGs).

Our results also suggest that facultative heterochromatin of hornworts is not only marked by H3K27me3 but also by H3K9me1 together with H3K27me1 on PCGs that are specifically expressed in sporophytes. Almost half of all TEs in *A*. *agrestis* are present in euchromatin or facultative heterochromatin, indicating a large degree of co‐option of TEs to PCGs and/or their regulatory sequences. Such co‐option has been reported mammals (Ninova *et al*., 2019) and in the pollen of *A*. *thaliana*, where reprogramming of H3K9me2 and DNA methylation is associated loci in vegetative nuclei promote expression of genes required for pollen tube growth (Borg *et al*., 2021; Khouider *et al*., 2021). The scattered location of constitutive heterochromatin in bryophytes contrasts with the position of heterochromatin around point centromeres in flowering plants with small genomes (Sigman & Slotkin, 2016; Naish *et al*., 2021). It has been proposed that heterochromatin participates in the recruitment of centromeric proteins in fission yeast, drosophila, and *A*. *thaliana* (Olszak *et al*., 2011; Scott & Sullivan, 2014). By contrast, bryophytes might have evolved mechanisms of centromere recruitment independent of constitutive heterochromatin and distinct from that of flowering plants. This mechanism could be related to the pervasive co‐option of TEs in bryophytes that selected their recruitment outside centromeres but not in discrete domains surrounding centromeres. The recent chromosome‐scale assembly of the genome of the streptophytic alga *Zygnema cf circumcarinatum* (Feng *et al*., 2023) shows an organization of TEs and PCGs similar to that of bryophytes. However, the massive diversification within the Zygnematophyceae (Hess *et al*., 2022), and the lack of knowledge of chromatin in this group, lycophytes, ferns, cycads, and gymnosperms currently prevents firmly concluding that the organization of chromatin in the first land plants and bryophytes were similar. The rapid extension of epigenomics in the evolutionary context of Zygnematophyceae (Hess *et al*., 2022) and new models of ferns (F.‐W. Li *et al*., 2018; Szövényi *et al*., 2021; Wickell *et al*., 2021; Kinosian & Wolf, 2022) will help to solve this question.

## Competing interests

None declared.

## Author contributions

TH and FB conceived and designed the experiments. TH performed ChIP‐seq and EM‐seq in *A*. *agrestis* with help from SA. SW annotated TEs in the genomes of *A*. *agrestis*. TH and SW performed the analysis of the data with help from EA who also performed statistical analyses and curated data. LD provided funding of SW and FB supervised the study. PS and F‐WL provided the genome assembly and gene annotations, HiC data and its analyses. TH and FB wrote the manuscript draft with input from all authors. TH and SW contributed equally to this work.

## Supporting information


**Dataset S1** Transposable element annotation of *Anthoceros agrestis*.

**Dataset S2** Homologs of *Marchantia polymorpha* sex chromosome genes.

**Dataset S3** Protein coding gene cluster assignment.

**Dataset S4** Gene Ontology term enrichment analyses per cluster.

**Dataset S5** Functional annotations of protein coding genes in cluster P2.

**Dataset S6** Transposable element cluster assignment.

**Fig. S1** Quality control of ChIP‐seq.
**Fig. S2** *K*‐means clustering of ChIP‐seq data over protein coding genes and transposable elements.
**Fig. S3** Expression level of protein coding genes in gametophyte and sporophyte.
**Fig. S4** DNA methylation levels over each transposable element family.
**Fig. S5** Distances between protein coding genes and transposable elements per cluster.
**Fig. S6** Genome browser view showing positional relationship between protein coding genes and transposable elements.

**Table S1** List of antibodies used in ChIP‐seq experiments.
**Table S2** Overlaps of peaks in each ChIP‐seq replicate.
**Table S3** Expression of genes encoding DNA methyltransferases.
Please note: Wiley is not responsible for the content or functionality of any Supporting Information supplied by the authors. Any queries (other than missing material) should be directed to the *New Phytologist* Central Office.

## Data Availability

Sequencing data generated in this study are available through NCBI Gene Expression Omnibus under the accession no. GSE218880 and NCBI Sequence Read Archive under the accession nos. SRR25338991, SRR10190639, SRR10190640, SRR10250248, SRR10250249, and SRR25366943. All code used in this study is available in https://github.com/wushyer/Anthoceros_chromatin_project.
