# Supplementary material for: The ancestral chromatin landscape of land plants
Source: New Phytol. 2023 Oct 12;240(5):2085–101. doi: 10.1111/nph.19311 (PMC10952607; doi:10.1111/nph.19311)
Supplement: Supplementary file 7 — Fig. S1 Quality control of ChIP‐seq. Fig. S2 K‐means clustering of ChIP‐seq data over protein coding genes and transposable elements. Fig. S3 Expression level of protein coding genes in gametophyte and sporophyte. Fig. S4 DNA methylation levels over each transposable element family. Fig. S5 Distances between protein coding genes and transposable elements per cluster. Fig. S6 Genome browser view showing positional relationship between protein coding genes and transposable elements. [file NPH-240-2085-s005.pdf]

***New Phytologist* Supporting Information**

The ancestral chromatin landscape of land plants

Authors: Tetsuya Hisanaga, Shuangyang Wu, Peter Schafran, Elin Axelsson, Svetlana Akimcheva, Liam Dolan, Fay-Wei Li, and Frédéric Berger

Article acceptance date: 29 August 2023

The following Supporting Information is available for this article:

Fig. S1 Quality control of ChIP-seq
Fig. S2 K-means clustering of ChIP-seq data over protein coding genes and transposable elements
Fig. S3 Expression level of protein coding genes in gametophyte and sporophyte
Fig. S4 DNA methylation levels over each transposable element family
Fig. S5 Distances between protein coding genes and transposable elements per cluster
Fig. S6 Genome browser view showing positional relationship between PCGs and TEs

See separate files for:

Table S1 List of antibodies used in ChIP-seq experiments
Table S2 Overlaps of peaks in each ChIP-seq replicate
Table S3 Expression of genes encoding DNA methyltransferases

Dataset S1 TE annotation of A. agrestis (GFF files can be opened using normal text editors.)
Dataset S2 Homologs of M. polymorpha sex chromosome genes
Dataset S3 PCG cluster assignment (BED files can be opened using normal text editors.)
Dataset S4 GOterm enrichment analyses per cluster
Dataset S5 Functional annotations of PCGs in cluster P2
Dataset S6 TE cluster assignment (BED files can be opened using normal text editors.)

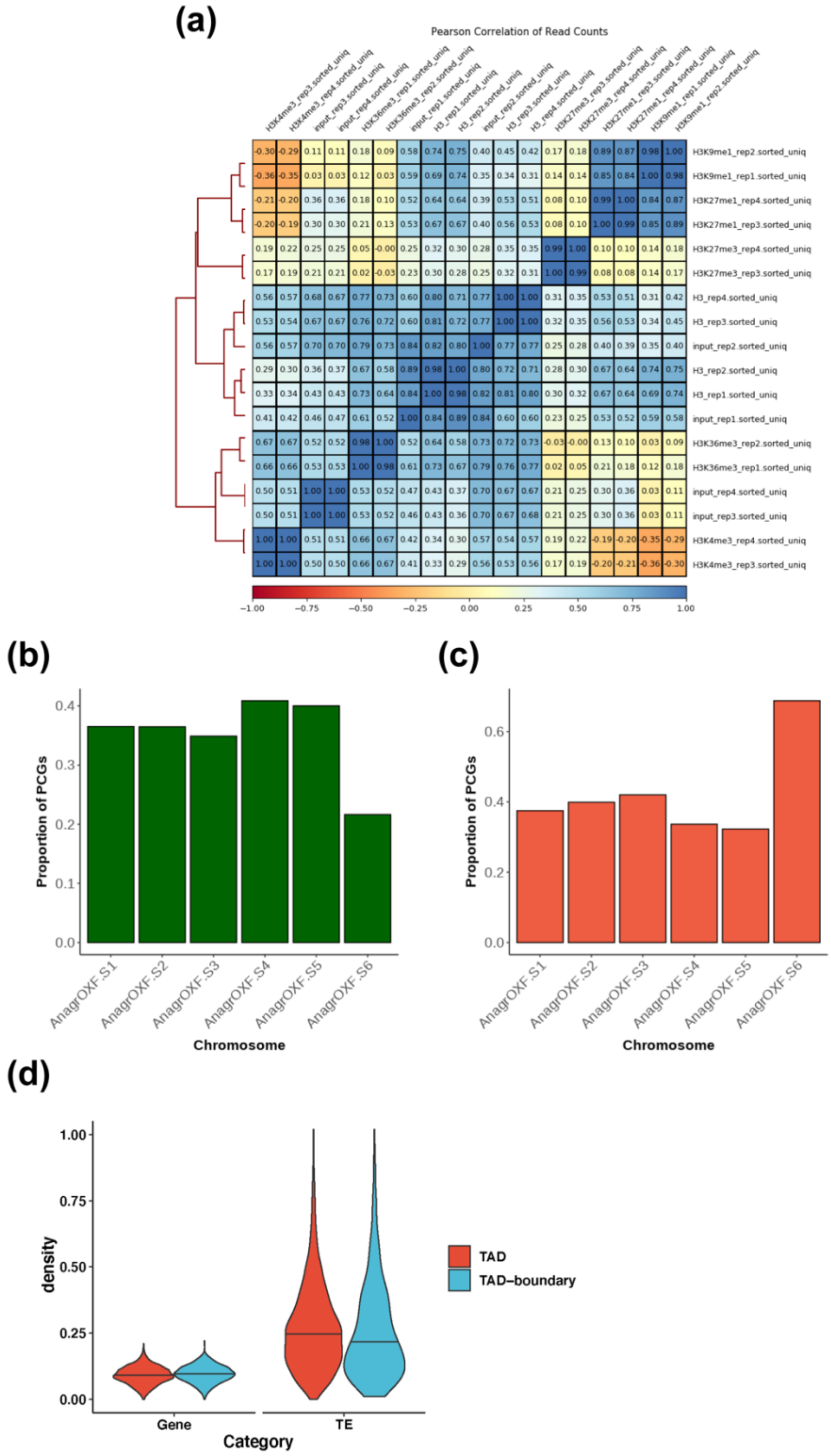

Fig. S1. Quality control of ChIP-seq

(a) Pearson correlation matrix showing that biological replicates of each mark cluster together.

(b) and (c) Bar plot showing the proportion of protein coding genes (PCGs, b) or transposable elements (TEs, c) in each chromosome. Proportions were calculated as the total length of the features in each chromosome is divided by the length of each chromosome.

(d) Violin plots showing density distribution of genomic features (PCGs or TEs) in topologically associated domains (TADs) and TAD boundaries. The density of genomic features was calculated as the number of features in each 40 kb window, divided by window length, and plotted for TADs and TAD boundaries. The median value is represented by a solid horizontal line.

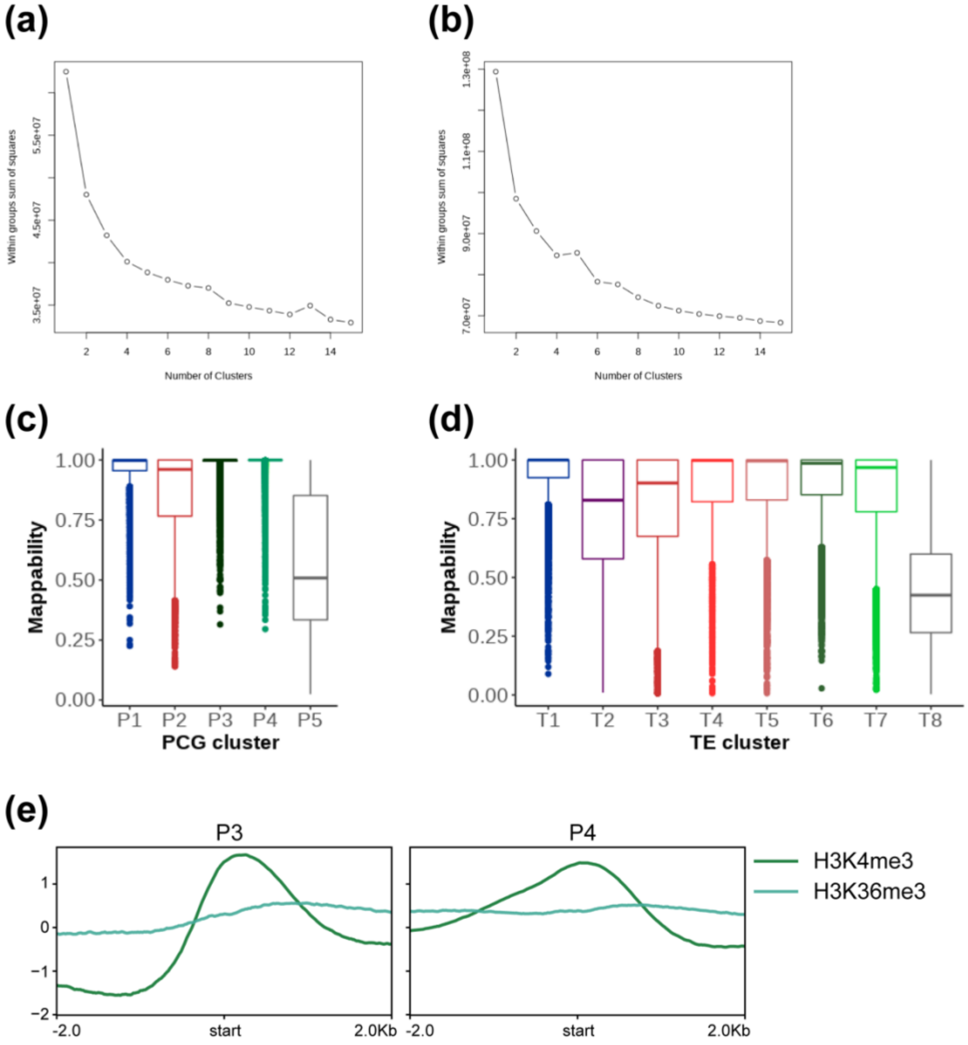

Fig. S2 . K-means clustering of ChIP-seq data over protein coding genes and transposable elements

(a) and (b) Within groups sum of squares calculated using the output files of computeMatrix command over protein coding genes (PCGs, a) or transposable elements (TEs, b) are plotted against numbers of clusters.

(c) and (d) boxplots indicating the mappability of PCGs (c) and TEs (d) per cluster. Median values are indicated by horizontal lines. Length of whiskers are based on the 1.5 x interquartile range value and values outside this range were treated as outliers and plotted by dots.

(e) Profile plot showing log2 ChIP/H3 enrichment of H3K4me3 and H3K36me3 over PCGs in clusters P3 and P4. Sequences 2 kb upstream and downstream of the start codon are included.

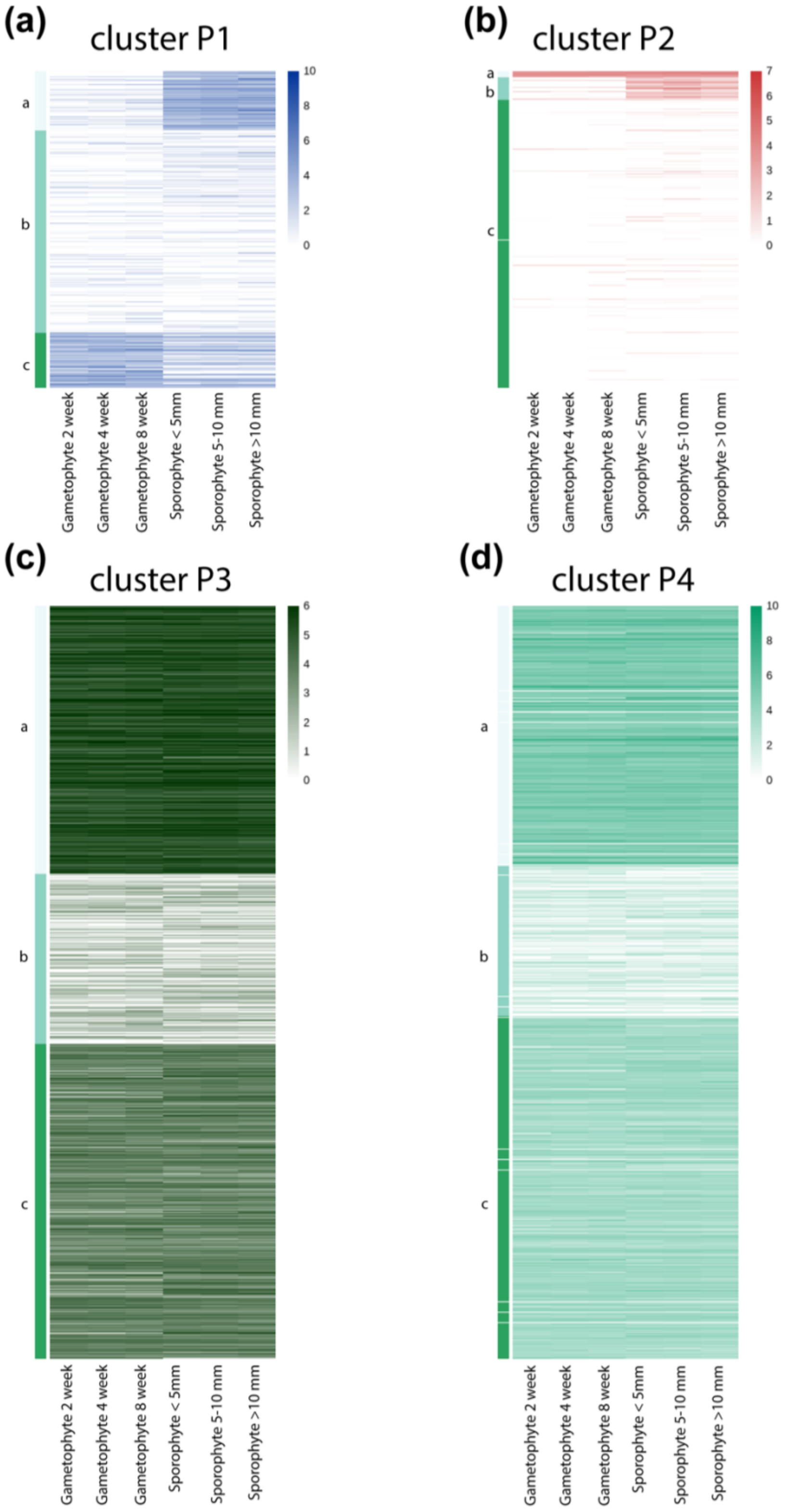

Fig. S3. Expression level of protein coding genes in gametophyte and sporophyte

(a), (b), (c) and (d) Heatmaps showing expression levels of protein coding genes in gametophyte and sporophyte tissue per cluster. Expression levels are indicated by arcsine Transcripts Per Million.

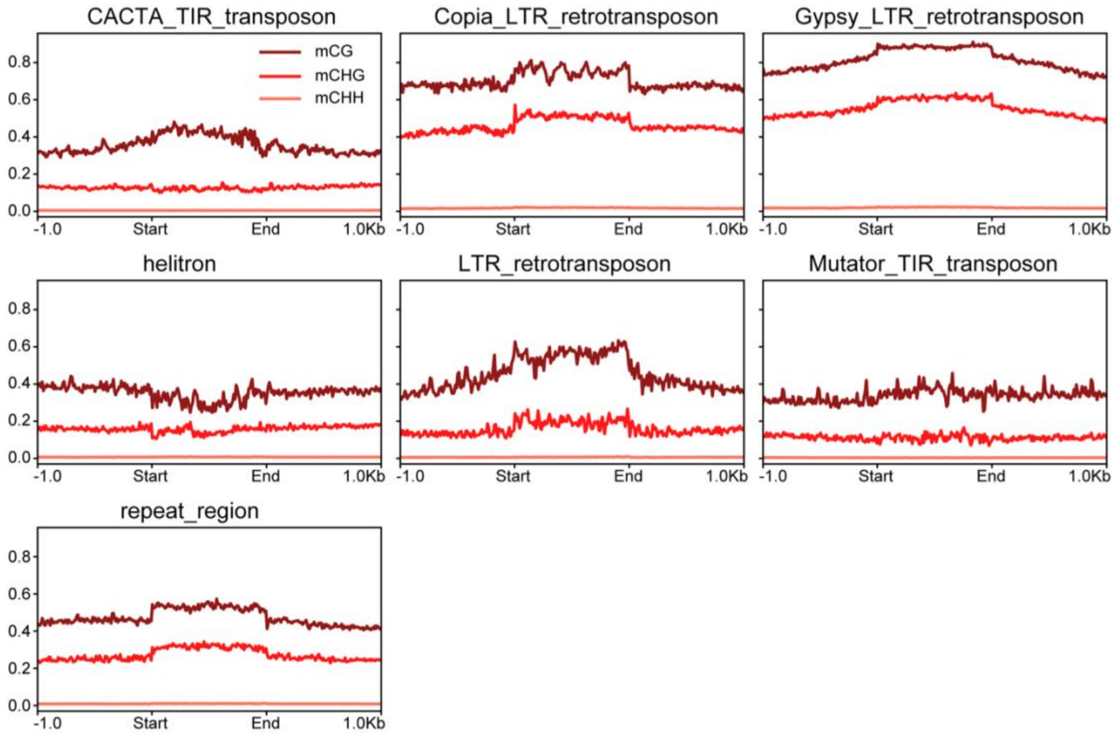

Fig. S4. DNA methylation levels over each transposable element family

Profile plot of CG, CHG, and CHH methylation levels over transposable elements (TEs) per TE family. Each TE annotation is scaled to 1 kb and sequences 1 kb upstream and downstream are included. Average methylation over 10 bp bins is plotted.

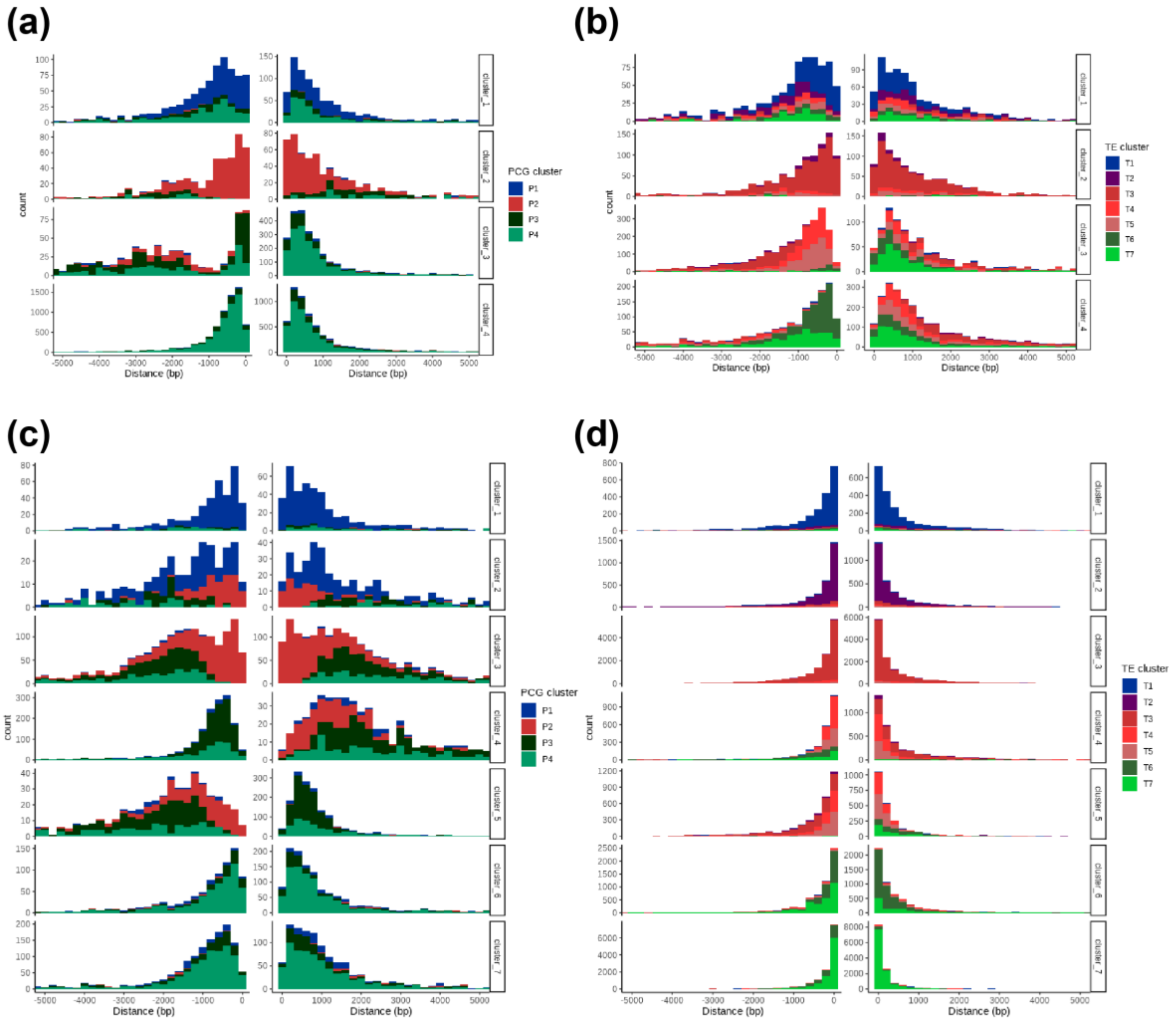

Fig. S5. Distances between protein coding genes and transposable elements per cluster

(a), (b), (c) and (d) Histograms showing distance from PCGs (a and b) or TEs (c and d) to the closest PCGs (a and c) or TEs (b and d) per cluster.

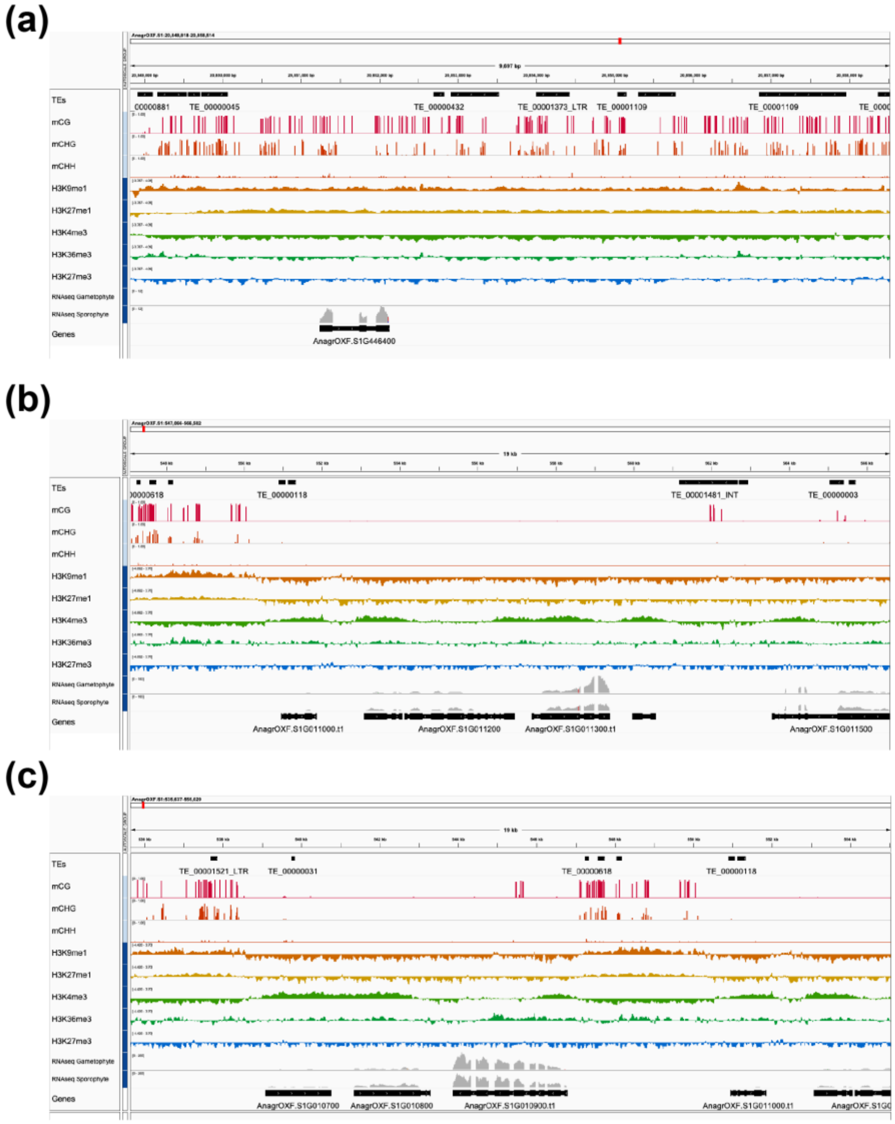

Fig. S6. Genome browser view showing positional relationship between PCGs and TEs

(a), (b) and (c) Integrative Genomics Viewer (IGV) browser screenshot demonstrating protein coding genes (PCGs) in the cluster P2 surrounded by transposable elements (TEs, a), PCGs in the cluster P4 forming a small euchromatic island (b) and TEs in the promoter region of PCGs in the cluster P3 (c). The regions shown are 9.5 kb in (a) or 19 kb in length in (b) and (c) from the scaffold AnagrOXF.S1. post-translational modification (PTM) tracks are bigwig files scaled by H3 coverage in 10-bp windows. DNA methylation tracks are bigwig files showing methylation levels of each cytosine site covered by at least 10 reads. ‘‘TEs’’ and ‘‘PCGs’’ tracks are annotation files for TEs and genes, respectively. ‘‘RNA-seq’’ tracks are bigwigs of mapped RNA-seq reads from

gametophyte tissue and sporophyte tissue (Li *et al.*, 2020). Scales are noted in square brackets in each track.
